# Feature Genes Selection Using Supervised Locally Linear Embedding and Correlation Coefficient for Microarray Classification

**DOI:** 10.1155/2018/5490513

**Published:** 2018-01-31

**Authors:** Jiucheng Xu, Huiyu Mu, Yun Wang, Fangzhou Huang

**Affiliations:** ^1^College of Computer and Information Engineering, Henan Normal University, Xinxiang 453007, China; ^2^Engineering Technology Research Center for Computing Intelligence and Data Mining, Henan Province 453007, China

## Abstract

The selection of feature genes with high recognition ability from the gene expression profiles has gained great significance in biology. However, most of the existing methods have a high time complexity and poor classification performance. Motivated by this, an effective feature selection method, called supervised locally linear embedding and Spearman's rank correlation coefficient (SLLE-SC^2^), is proposed which is based on the concept of locally linear embedding and correlation coefficient algorithms. Supervised locally linear embedding takes into account class label information and improves the classification performance. Furthermore, Spearman's rank correlation coefficient is used to remove the coexpression genes. The experiment results obtained on four public tumor microarray datasets illustrate that our method is valid and feasible.

## 1. Introduction

Cancer develops through either a series of genetic events or external influential factors that cause differential gene expression profile in the cancerous cells. The DNA microarray technology is pervasively used in the area of genomic research for diagnosing cancers [1]. Since the number of genes is typically larger than the number of samples, classification of microarray data is subjected to “the curse of dimensionality.” However, only a small number of genes are required in cancer diagnosis whereas the search space can be huge. Feature selection is an important step to reduce both dimension and redundancy (there is some obvious inaccuracy of gene expression in the experiment to obtain the gene expression data) of gene expression data during the classification process. According to the literature [2], the selection of feature genes methods is usually more important than developing classifier in the genomic data analysis. Therefore, how to choose the feature genes in gene expression profile effectively is the key point of bioinformatics study at present.

When mining in high-dimensional data, “the curse of dimensionality” is one of the major difficulties to overcome. The aim of feature selection is to reduce computational complexity while some desired inherent information of the data is conserved [3, 4]. Manifold learning is an ideal tool for machine learning that discovers the structure of high-dimensional data and gives better understanding of the data [5]. The representative of such methods comprises locally linear embedding (LLE), isometric mapping (Isomap), Laplacian eigenmaps (LE), and local tangent space alignment (LTSA) [6], and so on. In between, LLE is one of the most noted manifold learning methods and widely used in spectral analysis [7], edit propagation [8], fault detection [9, 10], image recognition [11, 12], and so on.

Subsequently, various improved LLE methods are designed to enhance the performance. Lai et al. [31] proposed a unified sparse learning framework by introducing the sparsity or L1-norm learning, which further extended the LLE-based methods to sparse cases. Theoretical connections between the orthogonal neighborhood preserving projection and the proposed sparse linear embedding are discovered. The ideal sparse embedding derived from the proposed framework is computed by iterating the modified elastic net and singular value decomposition. Cheng et al. [32] depended on the incremental locally linear embedding (ILLE) to improve the performance of fault-diagnosis for a satellite with high-dimensional telemetry data. Similarity, Liu et al. [33] put forward an incremental supervised LLE (I-SLLE) method for submersible plunger pump fault detection. In the I-SLLE algorithm, block matrix decomposition strategy is used to deal with out-of-sample data, while a part of original low-dimensional coordinates is also renovated, above which an iterative method is proposed to update all the dataset for improving the accuracy.

LLE has the advantage of global optimal solution of parsing without iteration. The low-dimensional embedding of calculation is summarized as sparse matrix eigenvalue calculation. So the complexity of calculation is relatively small. However, LLE mainly has the disadvantage of low self-learning ability and ignores the discriminant information. It is difficult to accurately capture the patterns on data and this could not gain higher effectiveness. Furthermore, the purpose of feature selection is to project the original data into a subspace with the following characteristics: the samples in the intraclass as close as possible and the samples in interclass far away from each other in the subspace. As mentioned before, feature genes selection distinguishes the pathogenic genes from normal genes. To solve this problem, de Ridder et al. extended the concept of LLE to multiple manifolds and proposed a supervised locally linear embedding (SLLE) algorithm which has been demonstrated to be a suitable feature for genes selection [34]. The dissimilarity between samples from different classes can be measured by metric function. It is commonly believed that the neighborhood of a sample in one class should consist of samples belonging to the same class. In the SLLE method, by taking into account class label information, the distance of interclass is larger than the Euclidean distance by adding a parameter to the pairs of points belonging to different classes. Otherwise, it remains as the Euclidean distance.

Feature selection reduces the dimension of feature and ensures the integrity of original dataset. It can improve the efficiency of data mining and dig out the results which are basically identical to the original dataset. More broadly, it is the problem of “the curse of the dimension.” However, the major consideration of SLLE is the relationship between the attributes and categories. The way to judge if an attribute is redundant is based on whether the attribute affects information discrimination of the class label. That is to say, SLLE remains not fully considered by the relationship between the attributes. In practice, it is not independent between the attributes, and there is a certain correlation between them. For instance, the dressing index and temperature are usually related: a high temperature means a low clothing index; otherwise the opposite occurs. It is inevitable that data redundancy will be caused by placing a large number of associated attributes in the reduction result. Correlation coefficient reflects the coexpression relationship between genes. The two genes are considered as coexpression when their correlation coefficient value is greater than a certain threshold; thus it can be removed [35, 36].

In order to solve the problem of poor classification performance in tumor classification, a novel feature genes selection method, called supervised locally linear embedding and Spearman's rank correlation coefficient (SLLE-SC^2^), is put forward in this paper. Supervised LLE algorithm, by taking into account class label information, is utilized to delete redundant genes. Meanwhile, Spearman's rank correlation coefficient is used to remove the coexpression genes. We also show biological investigation of the selected genes. Finally, we compared the performance of various classifiers based on the selected feature genes datasets. Results show that the SLLE-SC^2^ method selects a small set of nonredundant disease related genes with high specificity and achieves better efficiently compared with other related methods.

## 2. Research Methodology

### 2.1. Locally Linear Embedding

LLE approximates the input data with a low-dimensional surface and reduces its dimensionality by learning a mapping to the surface [37]. It first finds a group of the nearest neighbors of each data point. Then it calculates a set of weights for each data point that wonderfully describe the point as a linear combination of its neighbors. Finally, it finds the low-dimensional embedding of points by using an eigenvector-based optimization technique; thus each point is also described with the same linear combination of its neighbors. LLE is designed to establish such a feature mapping: low-dimensional embedding maintains the same local neighborhood relationship in high-dimensional space. It gets the corresponding low-dimensional embedding from the nearest neighbor graph of geometric properties in high-dimensional space under certain conditions. In fact, LLE considers the point of nearest neighbors, rather than distant points.

*(a) Assigning Neighbors to Each Data Point*. To find a group of nearest neighbors, LLE adopts *k* nearest neighbors (i.e., Euclidean distance) standard. Let *X* = {*x*_1_,…, *x*_*N*_} be a given dataset of *N* points, *x*_*i*_ ∈ *R*^*D*^; Euclidean distance is adopted to calculate the distance between samples *D*_*ij*_  (*i*, *j* ∈ 1,2,…, *n*) and find refactoring neighborhood of the *k* nearest neighbors for each data point.

*(b) Computing the Weights Best Linearly Reconstructed from Its Neighbors*. LLE computes the barycentric coordinates of a point *X*_*i*_ based on its neighbors *X*_*j*_. The original point is reconstructed by a linear combination and given by the weight matrix *W*_*ij*_ of its neighbors. Reconstruction errors are measured by the cost function

(1) εiW=min⁡Xi−∑j=1kWijXj2=min⁡∑jWijXi−Xj2=∑j,kWjWkGjk,

where *ε*_*i*_ is reconstruction error; *G*_*jk*_ is a local graham matrix.

(2) $$\begin{array}{c} G_{jk}={\left(\;X_{i}-X_{j}\;\right)}^{T}\left(\;X_{i}-X_{k}\;\right), \end{array}$$

where *G*_*jk*_ is a positive definite symmetric matrix. Equation (1) is a constrained least squares problem, and it is minimized under two constraints:

(3) Wij=1Xj  is  a  neighbor  of  Xi0the  others

(4) ∑jWij=1

in which, (3) is a constraint of coefficient. That is to say, each data point is reconstructed only from its neighbors. Equation (4) means the sum of every row of weight matrix equals 1. Thus (1) is rewritten as constrained optimization form:

(5) min ∑j,kWjWkGjks.t. ∑jWij=1.

Equation (5) is calculated by Lagrange multiplier approach. As *G*_*jk*_ is positive definite symmetric matrix, the inverse of the matrix *G*_*jk*_ exists. The optimal weight is calculated by

(6) $$\begin{array}{c} W_{j}=\frac{{\sum_{k}G_{jk}}^{-1}}{{\sum_{lm}G_{lm}}^{-1}}. \end{array}$$

*(c) Computing the Low-Dimensional Embedding Vector Best Reconstructed and Finding the Smallest Eigenmodes of the Sparse Symmetric Matric*. Each point *X*_*i*_ in the high-dimensional space is mapped onto a point *Y*_*i*_ in the low-dimensional space. The low-dimensional space *Y* is calculated by the following function:

(7) $$\begin{array}{c} \varepsilon \left(\;Y\;\right)=\min{\left\|\;\left.\;Y_{i}-\sum_{j=1}^{k}W_{ij}Y_{j}\;\right\|\;\right.}^{2}=\min\sum_{j,k}M_{ij}\left(\;Y_{i}\cdot Y_{j}\;\right). \end{array}$$

Cost function (7) is based on locally linear reconstruction errors, in which (*Y*_*i*_ · *Y*_*j*_) is inner product; *M*_*ij*_ is a sparse *N* × *N* matrix (*N* being the number of data points).

(8) $$\begin{array}{c} M_{ij}=\delta_{ij}-W_{ij}-W_{ji}+\sum_{k}W_{ki}W_{kj}, \end{array}$$

where *M*_*ij*_ is a positive definite symmetric matrix. Equation (7) is a minimization problem. Significantly, we can translate *Y*_*i*_ to any position without affecting the reconstruction error. Thus a constraint is added to eliminate the translational degree of freedom in (7). It requires all the center of low-dimensional embedding *Y*_*i*_ at the origin. Namely,

(9) $$\begin{array}{c} \sum_{i}Y_{i}=0. \end{array}$$

In order to eliminate the rotational and proportion degree of freedom, we add a constraint of unit covariance:

(10) $$\begin{array}{c} \frac{1}{n}*\sum_{i}Y_{i}Y_{i}^{T}=I; \end{array}$$

then (7) is regarded as a constrained optimization problem.

(11) min ∑j,kMijYi·Yjs.t. ∑iYi=0 1n·∑iYiYiT=I.

Equation (11) can be solved in multiple ways. One of the most effective methods is calculating cost matrix *M* relatively minimum *d* + 1 eigenvalue with its eigenvector which is optimized by using Lagrange multipliers. Notice that eigenvalue with its eigenvector is a fully 1 vector; it represents translation degrees of freedom corresponding to the 0 eigenvalue and requires removing. The retained *d* eigenvectors formed the output of LLE.

### 2.2. Supervised Locally Linear Embedding

LLE is an unsupervised manifold feature selection algorithm, which ignores the discriminant information of data. In order to improve the classification capability of LLE, discriminant information is assembled in the cost function of LLE (i.e., SLLE). SLLE is based on assumptions of the distance of data point from the same class less than the data point from the different classes and adds the discriminant information to the interclass distance. One of the solutions is to increase the Euclidean distance by adding a constant to the pairs of points from different classes, and the distance of data points from the same class is kept.

In a given set *X* = {*x*_1_, *x*_2_,…*x*_*n*_}, the distance metric is defined as

(12) $$\begin{array}{c} \Delta^{\prime }\left(\;i,j\;\right)=\Delta \left(\;i,j\;\right)+\lambda \cdot \max\left(\;\left\{\;\Delta \left(\;i,j\;\right)\;\right\}\;\right)\cdot \delta_{ij}, \end{array}$$

where Δ(*i*, *j*) is the Euclidean distance between *x*_*i*_ and *x*_*j*_. *λ* ∈ [0,1] is a tunable parameter. max⁡({Δ(*i*, *j*)}) is the maximum of Euclidean distance set {Δ(*i*, *j*)}. *δ*_*ij*_ is equal to 0 or 1 which is used to indicate whether the points belong to the same class; if *x*_*i*_ and *x*_*j*_ belong to the same class, *δ*_*ij*_ = 0; otherwise, *δ*_*ij*_ = 1.

It is worth noting that when *λ* = 0, the SLLE is turned into the original unsupervised LLE; when *λ* = 1, it is the supervised LLE; otherwise, it is a semisupervised LLE.

### 2.3. Spearman's Rank Correlation Coefficient

The relationship between attributes and categories relates to the feature reduction effectiveness and classification accuracy. Similarity, this connection is similar for attributes. In general, the connection between attributes is measured by correlation coefficient. The conventional measures of correlation coefficient are bivariate normal distribution, chi-square test for independence and rank correlation coefficient, and so on. Among them, Spearman's rank correlation coefficient is a nonparametric measure of rank correlation (statistical dependence between the ranking of two variables). It assesses how well is the relationship between two variables which is described with the monotonic function.

In a given dataset sample *X* = {*x*_1_, *x*_2_,…, *x*_*n*_}, attribute *C* = {*a*_1_, *a*_2_,…, *a*_*n*_}. The sequence *A*_*i*_ in sample *X*, relatively, attribute *a*_*i*_ with its attribute value is *A*_*i*_ = {*x*_1_ = *v*_1_, *x*_2_ = *v*_2_,…, *x*_*n*_ = *v*_*n*_}. Then the sequence *A*_*i*_ is sorted in descending order with rank for each sample (i.e., sample of the smallest attribute value with rank of 1, sample of the largest attribute value with rank of |*X*|; the rank takes an average with the attribute with the same value). Next, according to original sample order, we reorder the new rank sequence *A*′_*i*_ = {*x*_1_ = *v*′_1_, *x*_2_ = *v*′_2_,…, *x*_*n*_ = *v*′_*n*_}.

For the attributes *a*_*i*_, *a*_*j*_ of sample *k*, its rank sequence is *R*_*k*_ and *S*_*k*_, respectively. So we obtain |*U*| pairs rank combination (*R*_1_, *S*_1_), (*R*_2_, *S*_2_),…, (*R*|*U*|, *S*|*U*|). Spearman's rank correlation coefficient of attributes *a*_*i*_, *a*_*j*_ is defined as

(13) $$\begin{array}{c} r_{ij}=\left|\;r\left(\;a_{i},a_{j}\;\right)\;\right|=\frac{\sum_{k=1}^{\left|U\right|}\left[\left(R_{k}-\bar{R}\right)\left(S_{k}-\bar{S}\right)\right]}{\sqrt{\sum_{k=1}^{\left|U\right|}\left[{\left(R_{k}-\bar{R}\right)}^{2}{\left(S_{k}-\bar{S}\right)}^{2}\right]}}, \end{array}$$

where $\bar{R}=1/\left|U\right|*\sum_{k=1}^{\left|U\right|}R_{i}$, $\bar{S}=1/\left|U\right|*\sum_{k=1}^{\left|U\right|}S_{i}$. Correlation coefficient *r*_*ij*_ meets the following properties:

(1)  0 ≤ *r*_*ij*_ ≤ 1.

(2)*r*_*ij*_ always gives an answer between 0 and 1. The numbers in between are like a scale, where 1 indicates a very strong link and 0 indicates no link.

For more detailed instructions, we use an example to work out *r*_*ij*_ in Table 1. Sample *X* = {*x*_1_, *x*_2_, *x*_3_, *x*_4_, *x*_5_}; attribute *C* = {*a*_1_, *a*_2_}.

(1) Obtain the sequence *A*_1_ in sample *X*; relatively attribute *a*_1_ with its attribute value is *A*_1_ = {*x*_1_ = 0.7, *x*_2_ = 0.3, *x*_3_ = 0.5, *x*_4_ = 0.2, *x*_5_ = 0.8}.

(2) The sequence *A*_1_ is sorted in descending order with rank for each sample. Thus we obtain an ordered sequence of attribute {*x*_4_, *x*_2_, *x*_3_, *x*_1_, *x*_5_} and rank sequence {*x*_4_ = 1, *x*_2_ = 2, *x*_3_ = 3, *x*_1_ = 4, *x*_5_ = 5}.

(3) According to original sample order, we reorder the new rank sequence *R*_*a*_ = {*x*_1_ = 4, *x*_2_ = 2, *x*_3_ = 3, *x*_4_ = 1, *x*_5_ = 5}.

(4) In the same way, the rank sequence *S*_*a*_ in sample *X* relative attribute *a*_2_ with its attribute value is *A*_2_ = {*x*_1_ = 5, *x*_2_ = 2, *x*_3_ = 3, *x*_4_ = 1, *x*_5_ = 4}.

(5) The rank sequences *R*_*a*_ and *S*_*a*_ in sample *X* relatively attributed to its attribute value are shown in Table 2.

(6) Finally, according to (13), Spearman's rank correlation coefficient is 0.9 for this set of data.

### 2.4. Feature Genes Selection Using Supervised Locally Linear Embedding and Correlation Coefficient

Microarray data often contain redundant and noise features. These features could lead to poor classification performance and overfitting problems. Meanwhile, the gene expression data are in high-dimension and the number of feature gene datasets is very small which leads to the calculation falling into local optima and being computationally expensive. The key technique is to find a new feature genes selection method which can provide understanding and insight into tumor related cellular processes.

SLLE (by taking into account class label information) finds an ideal low-dimensional manifold of mapping for separating the intraclass and interclass. However, the main consideration of supervised algorithm is the relationship between the attributes and categories. That is to say, supervised learning algorithm is not fully considering the relationship between the attributes. In practice, the relationship between the attributes affects the reduction results and classification accuracy. It is inevitable that data redundancy will be caused by placing a large number of associated attributes in the reduction result. In general, the connection between attributes can be measured by correlation coefficient. Correlation coefficient reflects the coexpression relationship between genes. The two genes are considered as coexpression when their value of correlation coefficient is greater than a certain threshold; thus they are removed in feature genes selection. Spearman's rank correlation coefficient is a nonparametric measure of rank correlation (statistical dependence between the ranking of two variables).

Therefore we propose an effective SLLE-SC^2^ method for the selection of feature genes. Firstly, SLLE is used for reduction, mapping into the original data in a new feature space. Then considering the relationship between the attributes in the new feature space, Spearman's rank correlation coefficient is used for feature selection. Specifically, the PCA is used to compute the contribution of attributes, respectively, in the new feature space. Spearman's rank correlation coefficient is used to compute the maximum contribution of attribute and other attributes, respectively. If the value of correlation coefficient between attributes is greater than or equal to a preset threshold, the attribute is removed. Then loop is over the other attributes. SLLE method description is shown in Algorithm 1. Spearman's rank correlation coefficient method description is shown in Algorithm 2. Feature genes selection using SLLE-SC^2^ method description is shown in Algorithm 3.

## 3. Experiments and Results

### 3.1. Data Preparation

In order to verify the effectiveness of the proposed algorithm, four public tumor microarray datasets are used for making simulation experiment. Particularly, all of them represent binary classification tasks. Detailed information of datasets is shown in Table 3.

All numerical experiments are performed on a personal computer with 3.1 GHz AMD Athlon (tm) II and 4-G-byte memory. This computer runs Windows 7, with Matlab-R2010 and Weka-3.9.0.

### 3.2. Results and Analysis

In order to illustrate the reliability and comparability of tumor microarray datasets, we do experiment many times for the average. Experiments use 10-fold cross-validation. Specifically, based on preliminary tuning experiment, we set the nearest neighbors *k* for each data point as 5 for SLLE-SC^2^ method.

**Figure 1:**
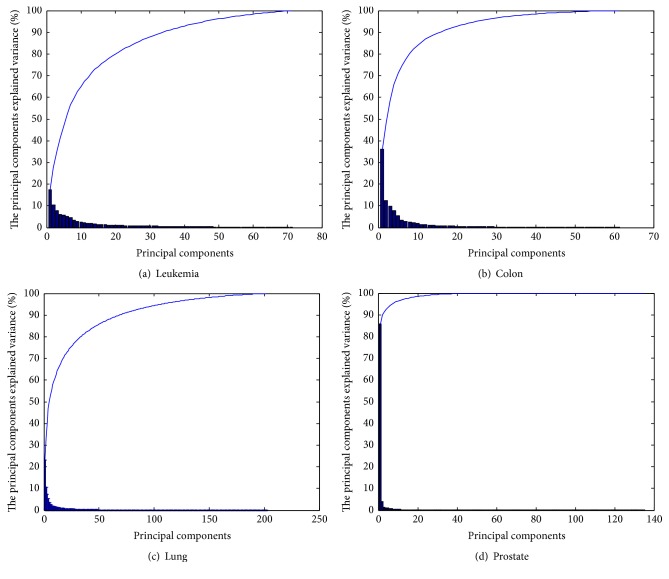
Pareto diagram of the principal components explained variance.

PCA algorithm is used for analyzing four tumor microarray datasets before SLLE-SC^2^ method test and drawing Pareto diagram (i.e., the information in genomic datasets) of the principal components explained variance for each dataset (blue curve said before the information content of total *n* genes in Figure 1). The results are shown in Figures 1(a), 1(b), 1(c), and 1(d).

The accumulation contribution rate of most datasets (except lung dataset) reaches more than 90 percent when the principal components of datasets are 50 (see Figure 1). It illustrates that gene expression profile datasets contain a large amount of redundancy (i.e., irrelevant and confounding factors) and the number of feature genes are a small part, so it is necessary to remove the redundancy genes.

**Figure 2:**
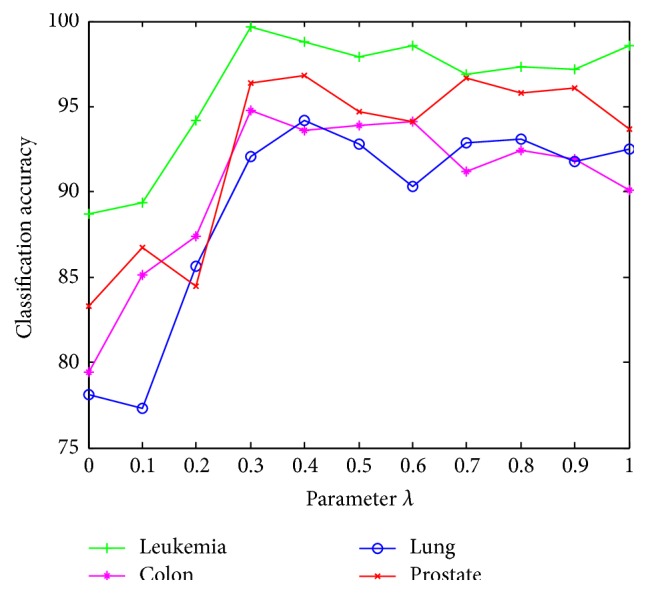
Classification accuracies with threshold *λ*.

The classification accuracies vary with the threshold *λ* of correlation coefficient; threshold *λ* takes values from 0 to 1 with step 0.1. For each value of the threshold, SLLE-SC^2^ obtains a subset of genes based on the average classification accuracies of SVM classifier. Experiments use 10-fold cross-validation. Classification accuracies with threshold *λ* are shown in Figure 2.

All the results show a common rule that the classification accuracies based on SVM increase with the value of threshold *λ* at first, arrive at a peak value, and then are stable relatively. It is easier for the classification of leukemia data than the others. When *λ* is among 0 to 0.3, classification accuracy increases faster, and when *λ* > 0.3, classification accuracy is relatively stable. It conforms to the actual performance. When *λ* is large, it has less strict requirements for removing redundant attributes, so the classification accuracy has no obvious change. Instead, when *λ* is small, it has many strict requirements for removing redundant attributes and causes decline of classification accuracy. For overall consideration, the threshold of correlation coefficient is 0.3.

For convenient description, the datasets in Table 3 are divided into positive and negative: positive ones are ALL and tumor, negative ones are AML and normal, respectively. TP and TN mean the number of right positive and negative examples; FN and FP denote the number of misclassified positive and negative examples, respectively.

(14) Acc=TP+TNTP+TN+FP+FNTPR=Recall=TPTP+FNTNR=TNTN+FPPrecision=TPTP+FPF-measure=2×Precision×RecallPrecision+RecallG-mean=TPR×TNR1/2.

(Note: Acc: overall accuracy; TPR: true positive rate; TNR: true negative rate; FPR: false positive rate; AUC: area under the receiver operating characteristic curve—it is the area below the ROC curve that depicts the performance of a classifier using the FPR and TPR pairs [38])

To present the superiority of SLLE-SC^2^ method, we evaluate it in comparison with that of SVM classification approaches and adopt the procedure of 10-fold cross-validation.  Table 4 reports the results of various performance metrics on four biomedicine datasets.

From the results in Table 4, our method with that of SVM classification results in better performance. Lung data acquire the lowest Acc value on all datasets. In terms of six important performance metrics, leukemia data obtain the largest Acc value, as well as taking the first place on four datasets for TNR, *F*-measure, and AUC criteria, respectively. In general, SLLE-SC^2^ algorithm gets a better effect in the aspects of high-dimensional and imbalanced classification tasks.

*(i) Classification Performance of Feature Genes. *Laplacian eigenmaps (LE), locally linear embedding (LLE), supervised locally linear embedding (SLLE), and Spearman's rank correlation coefficient (SC^2^) are implemented as competing methods to compare with the proposed SLLE-SC^2^ method. The nearest neighbor *k* is 5 for LE, LLE, SLLE, and SLLE-SC^2^. Four classifiers are implemented for classification including SVM, C4.5 (a classification algorithm of decision tree), Naive Bayes (naive Bayesian classification), and *k*-nearest neighbors (*k*NN). Experiments use 10-fold cross-validation; the results are shown in Tables 5–8.

Each result composes the classification accuracy of 20 independent outcomes in Tables 5–8. We see that SLLE-SC^2^ gains the greatest average accuracy in four datasets. By averaging across four classifiers, SLLE-SC^2^ obtains the top accuracy, with 100% (*k*NN classifier), 94.8% (Naive Bayes classifier), and 97.9% (SVM classifier) in the leukemia, lung, and prostate datasets, respectively. SC^2^ achieves the worst performance, and its accuracy is much lower than that of SLLE-SC^2^. SLLE by taking into account class label information gets much better classification performance.

*(ii) Comparison of the Classification Effect with the Gene Selected by Different Methods*. To verify classification effect with the gene selected by different methods, IGA-FBFE and other 9 feature selection methods are used for comparison in gene expression profiles. Lib-SVM classifier in Weka tool is used for simulation experiment. The number of feature genes and classification results are shown in Table 9.

As shown in Table 9, in terms of the number of selected genes, the difference between methods can be clearly found. For some methods, the number is as high as 60 (e.g., lung data with IGA-FBFE method) or even more, but for some methods the number is less than 10 (such as MAHP, SU, and SLLE-SC^2^ methods). However, it is hard to do a further comparison of the selected genes for the listed methods, as the genes selected by the other methods are not offered.

As for the classification accuracies, our method produces the results of 99.7% and 5 selected genes for the leukemia data. The results are not inferior to most of the published works. Colon data get small number of selected genes and higher accuracy. For lung data, ILasso and SU methods obtain better classification than our method but failure in number of feature genes. For prostate data, though BQPSO and IG-SGA acquire higher accuracy 99.25% and 100%, respectively, the number of feature genes is more than ours. Clearly, SLLE-SC^2^ cannot overcome all the existing methods. However, it can outperform some of the published methods and obtain a comparable result with most of the listed methods. Some of the methods produce high classification accuracy which use too large numbers of the selected genes in the classification (e.g., in prostate data, 26 genes are employed by IG-SGA method). However, such results may be difficult for a biological interpretation, all of which go to prove that our method selects the feature genes which have high classification ability and can reflect the structure of the data actuality. The small numbers of feature genes not only improve the running efficiency of the algorithm, but also can enhance the understanding of the microarray data.

*(iii) Biological Significance*. In order to validate the selected genes, Tables 10–13 summarize the index, gene, and description of the selected genes.

We search genes from the web of National Center for Biotechnology Information (NCBI) to further understand the selected genes (https://www.ncbi.nlm.nih.gov/). It can be seen that most of genes are closely associated with cancer as seen in Tables 10–13. Most of the selected genes are consistent with the results shown in the previous research [22–30]; for example, gene M23197 has been certified for targeted antibody therapy to make leukemia AML die [22], and the gene X95735 codes an LIM domain protein that is significant in cell adhesion of fibroblasts [23]. Gene AL050224 takes effect in the RNA polymerase and finds the overexpression in lung tissues [26]. Gene AJ011497 shows low-expression in MPM while showing high-expression in ADCA [27]. It is considered as a biomarker for the lung cancer. Gene M84526 codes another serine protease adipsin which is secreted by adipocytes into the bloodstream and functions as part of the alternative complement pathway of the innate immune system [29].

## 4. Conclusions

In this work, we explore the effects and benefits of SLLE-SC^2^ in the context of feature selection from high-dimensional genomic data. Specifically, supervised LLE is used to remove redundant genes. Considering the relationship between the attributes, the coexpression relationship between genes is deleted by Spearman's rank correlation coefficient. Our results on four microarray datasets are very promising and supported by existing biological knowledge. The results of our experiments give insight into both predominance and inferior position of SLLE-SC^2^ method and could represent a useful starting point to better understand the behavior of these techniques as well as the extent of their applicability to specific tumor problems. In more detail, we study genomic information to better understand pathogenesis of tumor and provide reference for the clinical treatment of tumor.

## Figures and Tables

**Algorithm 1:**
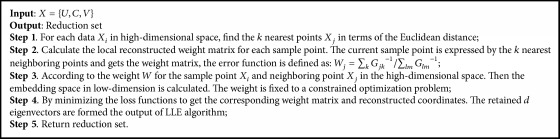
Supervised locally linear embedding method description.

**Algorithm 2:**
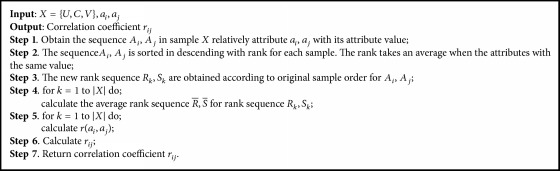
Spearman's rank correlation coefficient method description.

**Algorithm 3:**
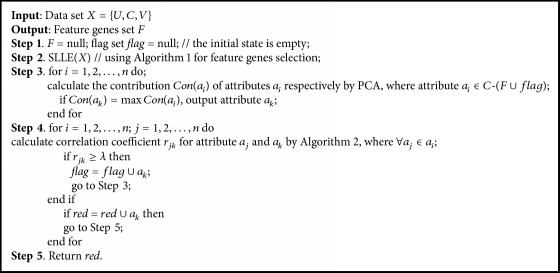
SLLE-SC^2^ method description.

**Table 1:** Example sample *X*.

Sample	*a*_1_	*a*_2_
*x*1	0.7	0.9
*x*2	0.3	0.3
*x*3	0.5	0.4
*x*4	0.2	0.1
*x*5	0.8	0.7

**Table 2:** The rank sequences *R*_*a*_ and *S*_*a*_.

Sample	*a*_1_	*R*_*a*_	*a*_2_	*S*_*a*_
*x*1	0.7	4	0.9	5
*x*2	0.3	2	0.3	2
*x*3	0.5	3	0.4	3
*x*4	0.2	1	0.1	1
*x*5	0.8	5	0.7	4

**Table 3:** Experiment dataset.

Dataset	Number of features	Classes	Number of instances
Leukemia	7129	ALL (47), AML (25)	72
Colon	2000	Tumor (40), normal (22)	62
Lung	12600	Tumor (186), normal (17)	203
Prostate	12600	Tumor (52), normal (50)	102

**Table 4:** The results of various performance metrics.

Dataset	Acc	TPR	TNR	*F*-measure	*G*-mean	AUC
Leukemia	0.997	0.86	0.882	0.909	0.895	0.914
Colon	0.948	0.89	0.877	0.85	0.911	0.864
Lung	0.942	0.793	0.827	0.842	0.837	0.858
Prostate	0.968	0.863	0.873	0.858	0.848	0.904

**Table 5:** Classification performance of leukemia data.

Classifiers	SLLE-SC^2^	LE	LLE	SLLE	SC^2^
SVM	99.7	85.9	92.3	97.4	85.2
C4.5	97.4	84.6	87.5	93.2	81.1
Naive Bayes	98.8	79.7	82.7	99.1	74.4
*k*NN	100	93.2	92.3	98.8	83.6

**Table 6:** Classification performance of colon data.

Classifiers	SLLE-SC^2^	LE	LLE	SLLE	SC^2^
SVM	94.8	81.2	89.1	91.9	80.5
C4.5	93.1	83.3	87.5	92.6	77.2
Naive Bayes	92.7	95.6	85.7	89.6	73.4
*k*NN	94.6	79.3	89.3	92.7	78.7

**Table 7:** Classification performance of lung data.

Classifiers	SLLE-SC^2^	LE	LLE	SLLE	SC^2^
SVM	94.2	80.5	87.1	91.6	80.6
C4.5	92.7	79.2	87.5	92.3	79.1
Naive Bayes	94.8	78.1	90.7	94.7	80.5
*k*NN	89.9	81.4	87.3	89.6	75.8

**Table 8:** Classification performance of prostate data.

Classifiers	SLLE-SC^2^	LE	LLE	SLLE	SC^2^
SVM	97.9	85.5	88.2	96.9	79.5
C4.5	95.4	81.3	90.7	95.3	81.1
Naive Bayes	94.8	79.1	86.7	89.9	73.7
*k*NN	96.8	82.9	87.3	97.8	74.8

**Table 9:** The number of feature genes and classification results.

Method	Leukemia	Colon	Lung	Prostate
IGA-FBFE [13]	94.20 (35)	90.09 (30)	91.23 (80)	88.12 (50)
BQPSO [14]	100 (7)	92.52 (11)	99.96 (9)	99.25 (10)
CAGC [15]	95.3 (866)	91.9 (135)	—	68.9 (3071)
ILASSO [16]	98.61 (14)	90.32 (4)	100 (7)	96.08 (9)
RT-PLSDA [17]	94.12 (9)	—	97.99 (4)	91.18 (18)
MAHP [18]	92.78 (5)	83.47 (5)	88.77 (5)	—
SU [19]	100 (6)	83.87 (4)	100 (3)	93.14 (4)
DRF0-CFS [20]	91.18 (13)	90.0 (10)	98.66 (17)	85.29 (113)
IG-SGA [21]	97.06 (3)	85.48 (60)	—	100 (26)
SLLE-SC^2^	99.7 (5)	95.4 (4)	94.8 (3)	97.3 (5)

**Table 10:** Biological significance of leukemia data.

Index	Gene selection	Description
1834	M23197	CD33 antigen (differentiation antigen) [22]
1882	M27891	CST3 cystatin C [22]
3847	U82759	GB DEF = homeodomain protein HoxA9 mRNA [23]
4847	X95735	Zyxin [23]
6041	L09209	APLP2 [22]

*Note. Index* denotes the serial number of the selected genes in the original data.

**Table 11:** Biological significance of colon data.

Index	Gene selection	Description
792	R88740	ATP synthase coupling factor 6, mitochondrial precursor [24]
1346	T62947	60S ribosomal protein l24 (*Arabidopsis thaliana*) [24]
1400	M59040	Human cell adhesion molecule (CD44) mRNA [25]
1772	H08393	Collagen alpha 2(xi) chain (*H. sapiens*) [24]

*Note. Index* denotes the serial number of the selected genes in the original data.

**Table 12:** Biological significance of lung data.

Index	Gene selection	Description
4336	AL050224	*Homosapiens* mRNA; cDNA DKFZp586L2123 [26]
7765	X05323	Human MOX2 gene for OX-2 membrane glycoprotein, exon 1, and joined CDS [27]
8537	AJ011497	*Homosapiens* mRNA for claudin-7 [27]

*Note. Index* denotes the serial number of the selected genes in the original data.

**Table 13:** Biological significance of prostate data.

Index	Gene selection	Description
5890	AJ001625	*Homosapiens* mRNA for Pex3 protein [28]
6462	M11433	Human cellular retinol-binding protein mRNA, complete cds [29]
9172	AI207842	Ao89h09.x1 *Homosapiens* cDNA, 3 ends [30]
9850	M84526	Human adipsin/complement factor D mRNA, complete cds [29]
12495	M98539	Human prostaglandin D2 synthase gene, exon 7 [29]

*Note. Index* denotes the serial number of the selected genes in the original data.
